# Supplementary material for: Impact hotspots of reduced nutrient discharge shift across the globe with population and dietary changes
Source: Nat Commun. 2019 Jun 14;10:2627. doi: 10.1038/s41467-019-10445-0 (PMC6570658; doi:10.1038/s41467-019-10445-0)
Supplement: Supplementary file 1 — Supplementary Information [file 41467_2019_10445_MOESM1_ESM.pdf]

Supplementary Information

Impact hotspots of reduced nutrient discharge shift across the globe with population and dietary changes

Wang et al.

*e-mail: xuwang@rcees.ac.cn or X.Wang@exeter.ac.uk.

Supplementary Figures and Tables

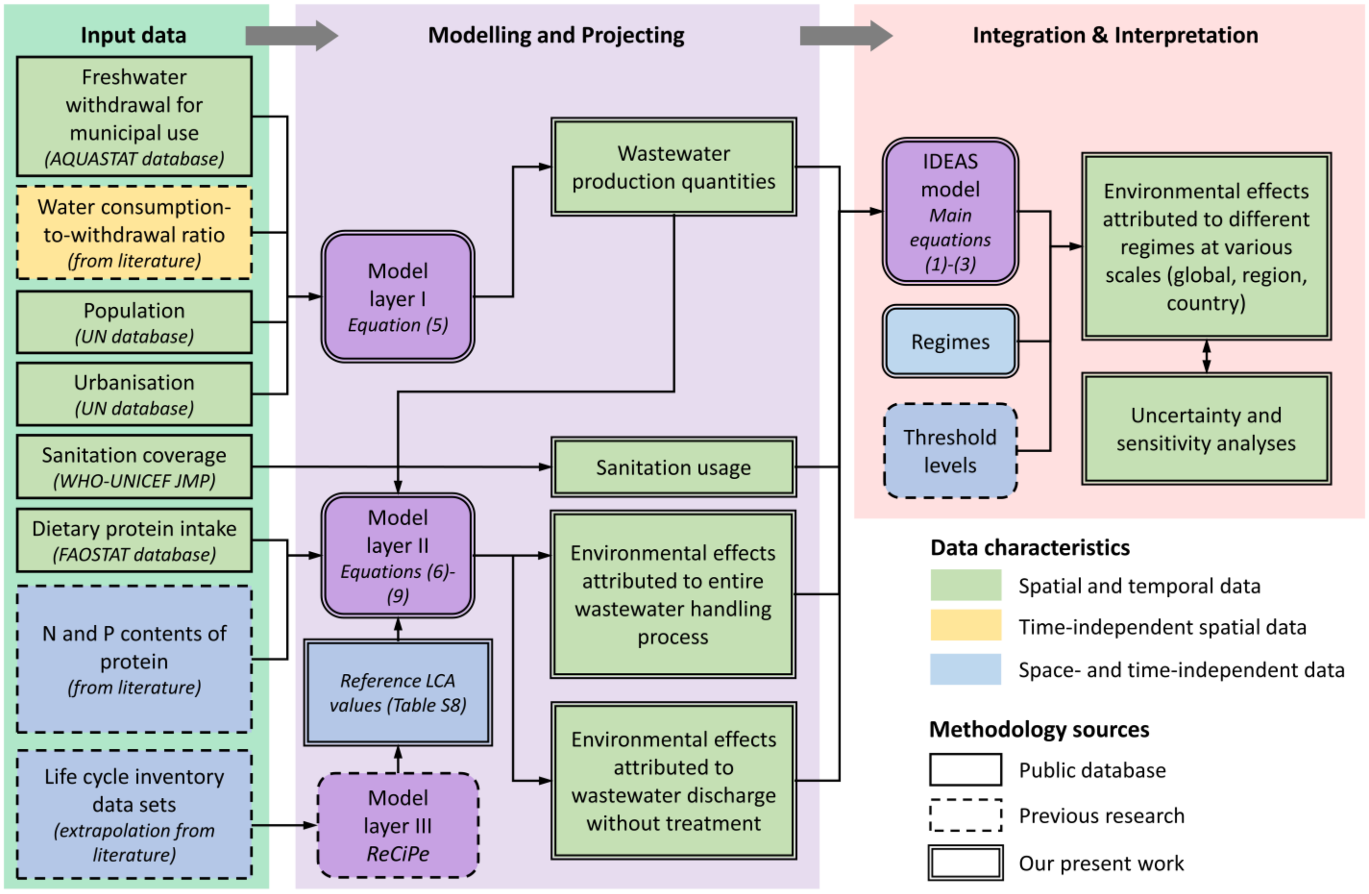

Supplementary Figure 1 | Scheme of the Integrating temporally and spatially variable demographic and socioeconomic Drivers to simulate the Environmental impacts of Alternative water and sanitation Services (IDEAS) model. Relevant tables, databases, and models are noted where appropriate.

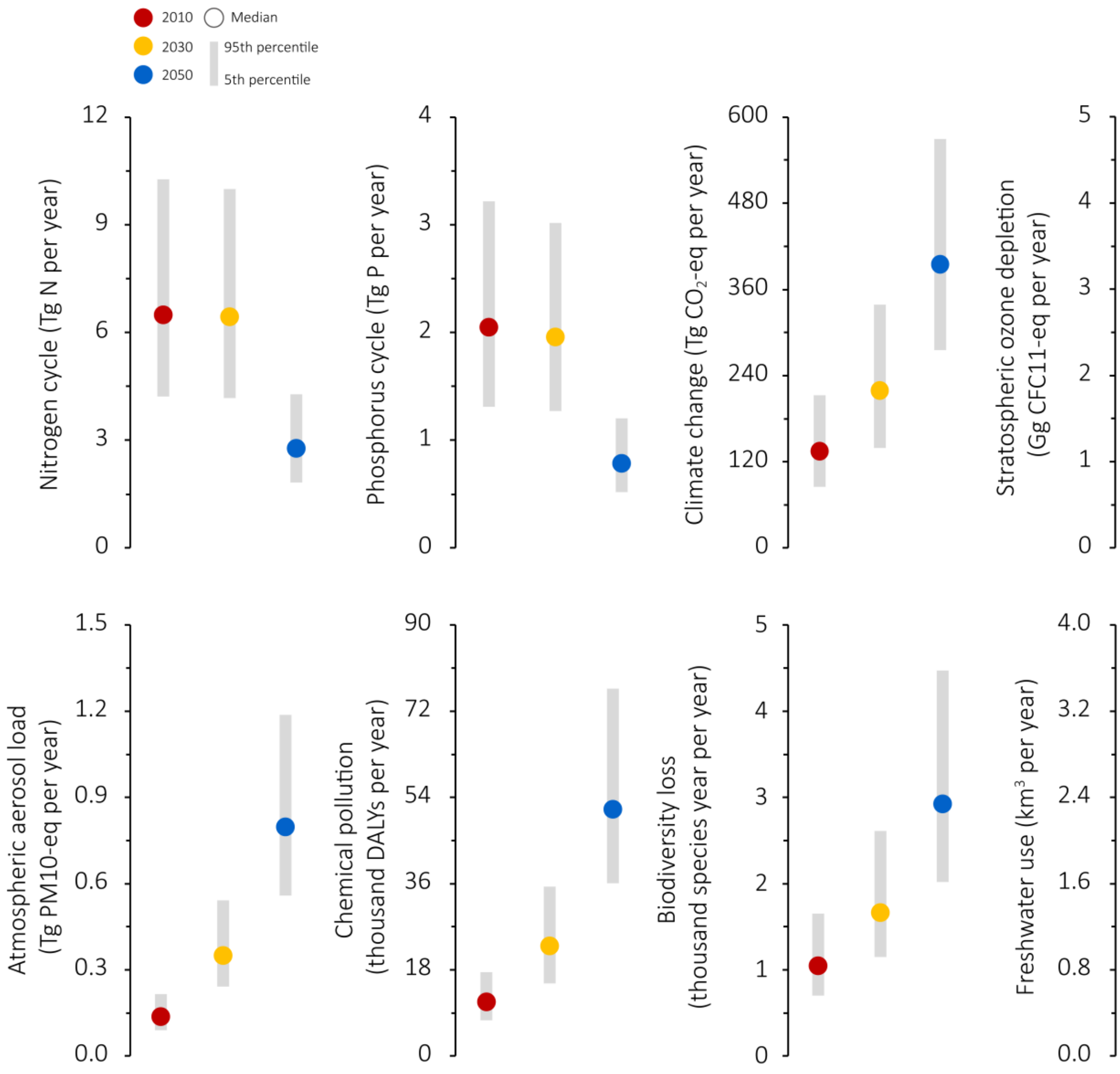

Supplementary Figure 2 | Global range values of the environmental impacts arising from wastewater management practices. Color circle is the global total of each environmental impact category presented in the main text (red, yellow, and blue represent the years 2010, 2030, and 2050, respectively). The criteria for including parameters in the uncertainty accounting is based on data quality assessment (Supplementary Table 4). The model parameters with worse data quality (average assessment value greater than 2) were included. The information regarding the model parameters used in this uncertainty analysis is presented in Supplementary Table 8. Error bars represent the 5th and 95th percentile values from the uncertainty analysis on each instance.

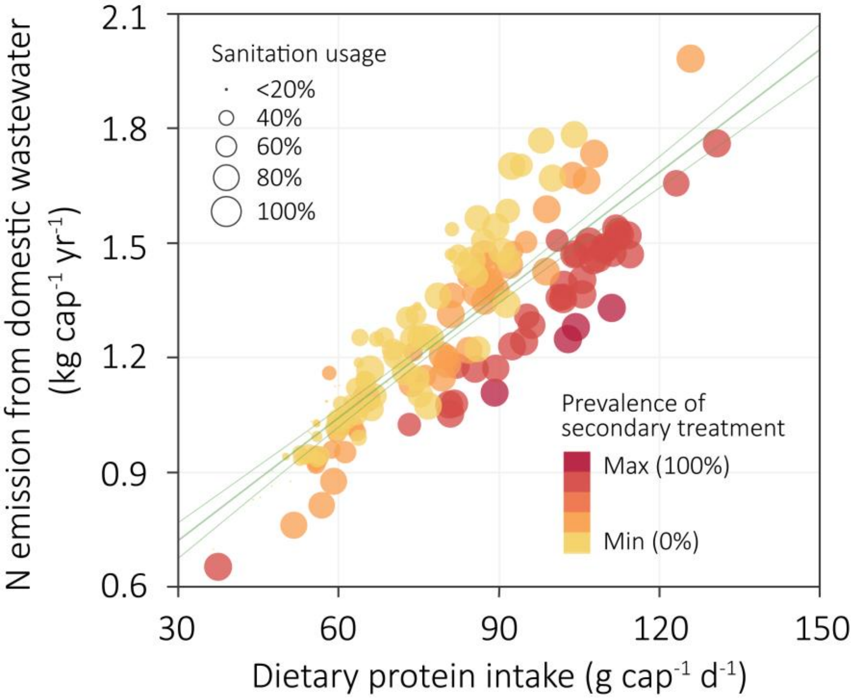

Supplementary Figure 3 | Correlations between per capita N emission from domestic wastewater, per capita dietary protein intake, sanitation usage, and prevalence of secondary wastewater treatment systems. Each circle represents mid-range data from one individual country in 2010 under the BAU regime, and 173 countries are included. Circle colors represent prevalence rates of secondary treatment facilities, while their sizes indicate sanitation usage. Green lines indicate the average trend (with 95% confidential intervals) in the relationship of per capita dietary protein intake and N emission from domestic wastewater (p<0.001, r^2=0.79). It can be observed that high per capital N emission is commonly correlated with intensive dietary protein intake, low usage of sanitation services, and small prevalence of secondary wastewater facilities, as well as combinations of these factors.

Supplementary Table 1 | Global, regional, and national mid-range N flows from domestic wastewater (Tg N yr^{-1}).

Regions and countries	BAU regime (2010)	STA regime (2030)	UTT regime (2050)
Global	8.7	8.6	3.7
Africa	1.2	1.7	1.8
western Africa	0.4	0.6	0.8
Asia	5.1	4.7	1.4
China	2.0	1.7	0.3
India	1.2	1.3	0.7
Americas	1.3	1.2	0.3
Oceania	<0.1 (0.04)	<0.1 (0.05)	<0.1 (0.02)
Europe	1.0	0.9	0.2

Supplementary Table 2 | The 173 countries considered in this analysis.

Continent	Region	Country[1]	Representativeness[2] Metric 1	Metric 2
Africa	Eastern	Burundi, Comoros, Djibouti, Eritrea*, Ethiopia, Kenya, Madagascar, Malawi, Mauritius, Mozambique, Rwanda, Somalia, South Sudan, Uganda, United Republic of Tanzania, Zambia, Zimbabwe	90%	100%
	Middle	Angola*, Cameroon, Central African Republic, Chad, Congo, Democratic Republic of the Congo, Equatorial Guinea, Gabon		
	Northern	Algeria, Egypt, Libya*, Morocco, Sudan, Tunisia		
	Southern	Botswana, Lesotho, Namibia, South Africa, Swaziland		
	Western	Benin, Burkina Faso, Cabo Verde*, Côte d'Ivoire, Gambia, Ghana, Guinea, Guinea-Bissau, Liberia, Mali, Mauritania, Niger, Nigeria, Senegal, Sierra Leone, Togo		
Americas	North	Canada*, United States of America*	64%	100%
	Caribbean	Barbados, Cuba, Dominica*, Dominican Republic, Grenada*, Haiti, Jamaica, Puerto Rico, Saint Kitts and Nevis*, Saint Lucia, Saint Vincent and the Grenadines, Trinidad and Tobago		
	Central	Belize, Costa Rica, El Salvador, Guatemala, Honduras, Mexico, Nicaragua, Panama		
	South	Argentina, Bolivia, Brazil, Chile*, Colombia, Ecuador, Guyana, Paraguay, Peru, Suriname, Uruguay, Venezuela*		
Asia	Eastern	China, Democratic People's Republic of Korea, Japan, Mongolia, Republic of Korea*	90%	99%
	South-central	Afghanistan, Bangladesh, Bhutan, India, Iran, Kazakhstan, Kyrgyzstan, Maldives, Nepal, Pakistan, Sri Lanka, Tajikistan, Turkmenistan*, Uzbekistan*		
	South-eastern	Cambodia, Indonesia, Lao People's Democratic Republic, Malaysia, Myanmar, Philippines, Singapore, Thailand, Timor-Leste, Viet Nam		
	Western	Armenia, Azerbaijan, Bahrain*, Cyprus, Georgia, Iraq, Israel, Jordan, Kuwait, Lebanon, Oman*, Qatar, Saudi Arabia, Syrian Arab Republic, Turkey, United Arab Emirates, Yemen		
Oceania	Australias	Australia, New Zealand*	17%	95%
	Melanesia	Fiji, Papua New Guinea		
Europe	Eastern	Czech Republic, Hungary, Poland, Republic of Moldova, Romania, Russian Federation, Slovakia, Ukraine	77%	98%
	Northern	Denmark, Estonia, Finland, Iceland, Ireland, Latvia, Lithuania, Norway, Sweden, United Kingdom		
	Southern	Albania, Bosnia and Herzegovina, Croatia, Greece, Italy, Malta, Montenegro, Portugal, Serbia, Spain		
	Western	Austria, Belgium, Bulgaria, France, Germany, Luxembourg, Monaco, Netherlands, Switzerland		

[1]In this analysis, 173 countries were chosen from 6 continents based on the availability of information and principle of consistency across data categories from 1990–2015. [2]Representativeness of the selected country set is assessed: Metric 1 is the ratio of the total number of the selected countries to the total of all countries related to a continent, while Metric 2 is the ratio of the total populations of the selected countries to the total populations of all countries related to a continent. Overall, the number of the selected countries accounts for over 70% of the global total, while the total populations of these countries make up 99% of the global population, thereby ensuring high representativeness of this analysis from a global perspective. *Improved sanitation includes facilities shared between two or more households.

Supplementary Table 3 | Country-level data sets used as inputs in this analysis*.

Data set	Unit	Time range of availability	Data source
Total population	-	1950–2015	UN Population Division[1]
Urban population	-	1950–2015	UN Population Division[1]
Rural population	-	1950–2015	UN Population Division[1]
Coverage of improved sanitation	% of population	1990–2015	WHO/UNICEF JMP[2]
Freshwater withdrawal for municipal use	m^3 $capital^{-1}$ yr^{-1}	1960–2015	AQUASTAT[3]
Total dietary protein intake	g $capital^{-1}$ d^{-1}	1961–2013	FAOSTAT[4]
Meat-based protein intake	g $capital^{-1}$ d^{-1}	1961–2013	FAOSTAT[4]
Plant-based protein intake	g $capital^{-1}$ d^{-1}	1961–2013	FAOSTAT[4]

*Includes the 173 countries listed in Supplementary Table 2. For added consistency across these different data sets, only data values during the period 1990–2010 were used for further analysis and extrapolation of future conditions.

Supplementary Table 4 | Results of the data quality assessment.							
**Category**	**Data acquisition**	**Data independence**	**Data representation**	**Data age**	**Geographical correlation**	**Technological correlation**	**Average value**
Total population	1	1	1	1	1	1	1.0
Urban population	1	1	1	1	1	1	1.0
Rural population	1	1	1	1	1	1	1.0
Coverage of improved sanitation	3	1	1	1	1	1	1.3
Freshwater withdrawal for municipal use	2	1	1	1	1	1	1.2
Total daily protein intake	2	1	1	1	1	1	1.2
Meat-based protein intake	2	1	1	1	1	1	1.2
Plant-based protein intake	2	1	1	1	1	1	1.2
N content of protein*	3	2	1	5	2	1	2.3
P content of meat-based protein*	3	2	1	5	2	1	2.3
P content of plant-based protein*	3	2	1	5	2	1	2.3
Water consumption to withdrawal ratio (urban areas)*	3	1	1	5	2	1	2.2
Water consumption to withdrawal ratio (rural areas)*	3	1	1	5	2	1	2.2
Reference LCA results*	3	2	1	3	2	5	2.7
Thresholds for planetary boundaries	3	1	1	2	1	1	1.5

The larger the assessment index, the worse is the data quality from the respective perspective. The values of each category are defined in Supplementary Table 5. *Data uncertainties are included in the accounting of uncertainty (Supplementary Figure 2).

**Supplementary Table 5 | Matrix used for the data quality assessment.**					
**Score**	**1**	**2**	**3**	**4**	**5**
Data acquisition	Measured data	Calculated data based on measurements	Calculated data partly based on assumptions	Qualified estimate by industrial expert	Non-qualified estimate
Data independence	Verified data, information from public or other independent source	Verified information from enterprises with interest in the study	Independent source, but based on non-verified information from industry	Non-verified information from industry	Non-verified information from the enterprise interested in this study
Data representation	Representative data from sufficient samples of sites over an adequate period	Representative data from smaller number of sites but for adequate periods	Representative data from adequate sites, but over shorter periods	Data from adequate number of sites but shorter periods	Representativeness unknown or incomplete data from smaller number of sites and/or from shorter periods, age unknown or more than 20 years of difference
Data age	Less than 3 years of difference to years of study	Less than 5 years of difference to years of study	Less than 10 years of difference to years of study	Less than 20 years of differences to year of study	Age unknown or more than 20 years of difference
Geographical correlation	Data from study area	Average data from larger area in which the study area is included	Data from area with similar production conditions	Data from area with slightly similar production conditions	Data from unknown area or with very different production conditions
Technological correlation	Data from enterprises, processes, and materials under study	Data from processes and materials under study but from different enterprises	Data from processes and materials under study but from different technology	Data on related processes or materials but same technology	Data on related processes or materials but different technology

A value of 1 means the best quality whereas a value of 5 reflects the worst quality. The assessment method refers to our previous study[5].

Supplementary Table 6 | Summary of the wastewater management regimes integrated with different value sets.

	BAU regime	STA regime	UTT regime
Time range	1990–2010	2011–2030	2031–2050
Typical year	2010	2030	2050
Spatial scale	173 countries	173 countries	173 countries
Population	UN medium-fertility total, urban, and rural estimates at country level[1]		
Urbanisation	Calculated ratios of the urban population to total population		
Daily dietary protein intake	Assumed to follow FAO estimates[4] for per capita daily protein intake from total food, meat- and plant-based food where possible; else linear extrapolation with negative slope replacement		
N and P contents of protein	Assumed a value of 0.13 to the ratio, noting the N content of protein, while assumed values of 0.011 and 0.022 to the ratios for estimating the P contents of meat- and plant-based protein, respectively[6]		
Water withdrawal for municipal use	Assumed to follow AQUASTA estimates[4] where possible; else linear extrapolation with negative slope replacement		
Water consumption	Assumed the water consumption to withdrawal ratio to a median level of 0.1 and 0.3 for urban and rural areas[7], respectively		
Sanitation coverage	Assumed to directly use the historical data from 1990 to 2010 provided in the database[2]	Linear extrapolation based on data[2] from 1990 to 2010; with negative slope replacement	
Prevalence of wastewater treatment	Secondary treatment[8]: 0% (Africa), 14% (Americas excl. North America), 90% (North America), 35% (Asia), 0% (Oceania excl. Australia), 90% (Australia), 66% (Europe). The percentages of the wastewater infrastructure served by secondary treatment are subtracted from 100% to estimate the fractions of the facilities served by primary treatment.	Universal access to secondary treatment (100% globally)	Universal access to tertiary treatment (100% globally)
Nutrient removal efficiency*	Primary treatment: 10% for N and P; secondary treatment: 40% for N and 45% for P	Secondary treatment: 40% for N and 45% for P	Tertiary treatment: 90% for N and 95% for P
Treatment approach	Various typical wastewater treatment approaches for nutrient removal are included, with regime-specific design and operational parameters (Supplementary Table 7)		

*The nutrient removal efficiencies for N and P are summarised from the literature[9, 10, 11, 12].

Supplementary Table 7 | Summary of various wastewater treatment approaches included in this analysis.

Wastewater treatment level	Nutrient removal efficiency	Main wastewater treatment approaches	General note
Primary	10% for both N and P	Primary sedimentation	(i) The life cycle inventory data (including from construction and operational phases) in relation to the different wastewater treatment approaches derive mainly from the literature[13]. (ii) Although similar approaches were utilised to treat nutrients in both secondary and tertiary treatments in this study, life cycle inventory data of these approaches varied with different nutrient removal demands. (iii) Range values from the above-mentioned literature are used in a ReCiPe model to acquire the reference LCA results under different wastewater treatment levels (Supplementary Table 8).
Secondary	40% for N and 45% for P	Modified Ludzack-Ettinger, oxidation ditch, 5-stage Bardenpho, 4-stage membrane bioreactor, Anaerobic membrane bioreactor plus 5-stage Bardenpho	
Tertiary	90% for N and 95% for P	Oxidation ditch, 5-stage Bardenpho, 4-stage membrane bioreactor, Anaerobic membrane bioreactor plus 5-stage Bardenpho, and combinations	

Supplementary Table 8 | Distribution of the parameters used in uncertainty analyses of each regime[1].

Parameter			Value	Range	Distribution	Source
N content of protein (%)			13	13 – 19	Uniform	Literature[6]
P content of plant-based protein (%)			2.2	0.4 – 4.8	Triangular	Literature[6]
P content of meat-based protein (%)			1.1	0.2 – 3.2	Triangular	Literature[6]
Water consumption to withdrawal ratio (%)		Urban	10	5 – 15	Triangular	Literature[7]
		Rural	30	10 – 50	Triangular	Literature[7]
Reference LCA results[2]	Without treatment	NC (kg N m^{-3})	5.00×10^{-2}	4.96×10^{-2} – 5.02×10^{-2}	Triangular	Calculations based on life cycle inventory data and assumptions from literature[5, 12, 13, 14]
		PC (Kg yr P m^{-3})	1.20×10^{-2}	1.19×10^{-2} – 1.22×10^{-2}	Triangular	
		CC (kg CO_2-eq m^{-3})	0.37	0.35 – 0.66	Triangular	
		OD (kg CFC11-eq m^{-3})	3.60×10^{-6}	3.58×10^{-6}–3.61×10^{-6}	Triangular	
		AL (kg PM10-eq m^{-3})	6.85×10^{-7}	6.84×10^{-7} – 6.86×10^{-7}	Triangular	
		CP (DALYs m^{-3})	1.69×10^{-8}	1.67×10^{-8} – 1.71×10^{-8}	Triangular	
		BL (species yr yr^{-1} m^{-3})	3.01×10^{-9}	3.00×10^{-9} – 3.02×10^{-9}	Triangular	
		FU (m^3 m^{-3})	1.37×10^{-5}	1.36×10^{-5} – 1.38×10^{-5}	Triangular	
	Primary treatment	NC (kg N m^{-3})	4.50×10^{-2}	4.46×10^{-2} – 4.56×10^{-2}	Triangular	
		PC (Kg yr P m^{-3})	1.08×10^{-2}	1.07×10^{-2} – 1.09×10^{-2}	Triangular	
		CC (kg CO_2-eq m^{-3})	0.62	0.56 – 0.73	Triangular	
		OD (kg CFC11-eq m^{-3})	5.97×10^{-6}	5.96×10^{-6} – 5.98×10^{-6}	Triangular	
		AL (kg PM10-eq m^{-3})	6.67×10^{-4}	5.81×10^{-4} – 8.21×10^{-4}	Triangular	
		CP (DALYs m^{-3})	4.17×10^{-8}	3.64×10^{-8} – 5.03×10^{-8}	Triangular	
		BL (species yr yr^{-1} m^{-3})	4.76×10^{-9}	4.25×10^{-9} – 5.68×10^{-9}	Triangular	
		FU (m^3 m^{-3})	9.80×10^{-4}	7.44×10^{-4} – 1.19×10^{-3}	Triangular	
	Secondary treatment	NC (kg N m^{-3})	3.02×10^{-2}	2.99×10^{-2} – 3.07×10^{-2}	Triangular	
		PC (Kg yr P m^{-3})	6.73×10^{-3}	6.62×10^{-3} – 6.89×10^{-3}	Triangular	
		CC (kg CO_2-eq m^{-3})	1.01	0.92 – 1.17	Triangular	
		OD (kg CFC11-eq m^{-3})	9.01×10^{-6}	9.00×10^{-6} – 9.15×10^{-6}	Triangular	
		AL (kg PM10-eq m^{-3})	1.75×10^{-3}	1.56×10^{-3} – 2.09×10^{-3}	Triangular	
		CP (DALYs m^{-3})	1.11×10^{-7}	1.05×10^{-7} – 1.23×10^{-7}	Triangular	
		BL (species yr yr^{-1} m^{-3})	7.55×10^{-9}	6.78×10^{-9} – 8.96×10^{-9}	Triangular	
		FU (m^3 m^{-3})	4.55×10^{-3}	4.20×10^{-4} – 5.17×10^{-3}	Triangular	
	Tertiary treatment	NC (kg N m^{-3})	5.45×10^{-3}	5.40×10^{-3} – 5.50×10^{-3}	Triangular	
		PC (Kg yr P m^{-3})	8.62×10^{-4}	8.60×10^{-4} – 8.65×10^{-4}	Triangular	
		CC (kg CO_2-eq m^{-3})	1.61	1.43 – 1.88	Triangular	
		OD (kg CFC11-eq m^{-3})	1.37×10^{-5}	1.36×10^{-5} – 1.38×10^{-5}	Triangular	
		AL (kg PM10-eq m^{-3})	3.39×10^{-3}	2.71×10^{-3} – 4.38×10^{-3}	Triangular	
		CP (DALYs m^{-3})	2.16×10^{-7}	1.83×10^{-7} – 2.58×10^{-7}	Triangular	
		BL (species yr yr^{-1} m^{-3})	1.18×10^{-8}	1.03×10^{-8} – 1.40×10^{-8}	Triangular	
		FU (m^3 m^{-3})	9.99×10^{-3}	7.28×10^{-3} – 1.36×10^{-2}	Triangular	

[1]Mid-range, minimal, and maximal values are calculated based on the life cycle inventory data and determination approaches from the literature[5, 13, 14]. [2]NC, PC, CC, OD, AL, CP, BL, and FU represent nitrogen cycle, phosphorus cycle, climate change, stratospheric ozone depletion, atmospheric aerosol loading, chemical pollution, biodiversity loss, and freshwater use, respectively.

Supplementary References

1. UN. *World Urbanization Prospects: The 2014 Revision.* (United Nations, Department of Economic and Social Affairs, Population Division, 2015).
2. WHO-UNICEF. *Joint Monitoring Programme for Water Supply and Sanitation: Estimates on the use of water sources and sanitation facilities.* (World Health Organization, 2015).
3. AQUASTAT - Food and Agriculture Organization's Information System on Water and Agriculture. Available at www.fao.org/nr/aquastat/ (accessed on 1 March 2016).
4. FAOSTAT. Food and Agriculture Organization statistics division. Available at http://www.fao.org/faostat/en/ (accessed on 1 March 2016).
5. Wang X., Liu J. X., Ren N.-Q., Yu H. Q., Lee D. J. & Guo X. S. Assessment of multiple sustainability demands for wastewater treatment alternatives: A refined evaluation scheme and case study. *Environ. Sci. Technol.* **46**, 5542-5549 (2012).
6. Jönsson H., Richert Stintzing A., Vinnerås B. & Salomon E. *Guidelines on use of urine and faeces in crop proudction* (Stockholm Environment Institute, 2004).
7. Ritchie H. & Roser M. Water Access, Resources & Sanitation (Published online and availabe at http://ourworldindata.org/water-access-resources-saniation/, 2017).
8. WHO/UNICEF. *Global Water Supply and Sanitation Assessment 2000 Report.* (World Health Organization, 2000).
9. Wang X. H., Wang X., Huppes G., Heijungs R. & Ren N. Q. Environmental implications of increasingly stringent sewage discharge standards in municipal wastewater treatment plants: Case study of a cool area of China. *J. Clean. P.* **94**, 278-283 (2015).
10. Van Drecht G., Bouwman A.F., Knoop J.M., Beusen A.H.W., Meinardi C.R. Global modeling of the fate of nitrogen from point and nonpoint sources in soils, groundwater, and surface water. *Global Biogeochem. Cy.* **17**, (2003).
11. Schaubroeck T., *et al.* Environmental sustainability of an energy self-sufficient sewage treatment plant: Improvements through DEMON and co-digestion. *Water Res.* **74**, 166-179 (2015).
12. Bradford-Hartke Z., Lane J., Lant P. & Leslie G. Environmental benefits and burdens of phosphorus recovery from municipal wastewater. *Environ. Sci. Technol.* **49**, 8611-8622 (2015).
13. Foley J., de Haas D., Hartley K. & Lant P. Comprehensive life cycle inventories of alternative wastewater treatment systems. *Water Res.* **44**, 1654-1666 (2010).
14. Wang X. *et al.* Evolving wastewater infrastructure paradigm to enhance harmony with nature. *Sci. Adv.* **4**, eaaq0210 (2018).
