# Supplementary material for: Impact hotspots of reduced nutrient discharge shift across the globe with population and dietary changes
Source: Nat Commun. 2019 Jun 14;10:2627. doi: 10.1038/s41467-019-10445-0 (PMC6570658; doi:10.1038/s41467-019-10445-0)
Supplement: Supplementary file 3 — Description of Additional Supplementary Files [file 41467_2019_10445_MOESM3_ESM.docx]

Description of Additional Supplementary Files

File Name: Supplementary Dataset 1

Description: Estimating the N and P flows for the three wastewater management regimes

File Name: Supplementary Dataset 2

Description: Normalising the broad environmental impacts arising from wastewater management practices

File Name: Supplementary Dataset 3

Description: Complete sensitivity analysis of the model outputs.
